# A New System for the Rapid Collection of Large Numbers of Developmentally Staged Zebrafish Embryos

**DOI:** 10.1371/journal.pone.0021715

**Published:** 2011-06-29

**Authors:** Isaac Adatto, Christian Lawrence, Michael Thompson, Leonard I. Zon

**Affiliations:** 1 Stem Cell Program and Division of Hematology/Oncology, Children's Hospital and Dana Farber Cancer Institute, Boston, Massachusetts, United States of America; 2 Harvard Stem Cell Institute, Boston, Massachusetts, United States of America; 3 Harvard Medical School, Boston, Massachusetts, United States of America; 4 Howard Hughes Medical Institute, Boston, Massachusetts, United States of America; 5 Aquatic Resources Program, Children's Hospital Boston, Boston, Massachusetts, United States of America; 6 Plastics Concepts, Inc., Billerica, Massachusetts, United States of America; Hong Kong University of Science and Technology, China

## Abstract

The zebrafish is an excellent genetic and developmental model system used to study biology and disease. While the zebrafish model is associated with high fecundity, its reproductive potential has not been completely realized by scientists. One major issue is that embryo collection is inefficient. Here, we have developed an innovative breeding vessel designed to stimulate the natural reproductive behavior of the fish. This novel apparatus allows us to collect large numbers of developmentally synchronized embryos in brief and defined windows of time, and with minimal investments in labor and space. To demonstrate the efficacy of this approach, we placed three separate groups (n = 180) of fish in the vessel and allowed them to spawn for 10-minute intervals. During these trials, which were repeated three times, the fish produced 8600±917, 8400±794, and 6800±1997 embryos, respectively. This level of embryo production is nearly twice what we were able to achieve when using conventional crossing equipment with some of the same fish, and it required significantly less room and time to set up and break down. This system overcomes major space and labor restrictions inherent in spawning equipment currently used in the field, and will greatly accelerate efforts to improve the scale and throughput of experiments.

## Introduction

A number of features make the zebrafish (*Danio rerio*) an excellent experimental subject, particularly its high fecundity. A healthy, sexually mature female fish is capable of producing hundreds of offspring every day, and individual clutch sizes may exceed 700 eggs[1]. This tremendous reproductive potential makes the zebrafish embryo/larva particularly suitable for use in studies where high rate of throughput and/or automation are advantageous. The methods and equipment typically used to collect newly spawned zebrafish embryos in the laboratory do not allow this potential to be fully realized. The most common approach involves placing a small (typically 1–2 L) polycarbonate mating cage or insert with a mesh bottom inside a slightly larger container that is filled with water. Pairs of males and females or small mixed-sex groups (typically 5 fish total) are then added to the mating cage on the evening prior to the morning when embryos are desired. Male and female fish may be separated overnight by means of a small divider. The following morning, the divider is removed, allowing the fish to spawn. Newly fertilized embryos fall through the mesh “floor” of the insert to facilitate collection while protecting them from cannibalization by adults [2], [3].

While this technique is generally effective, the amount of time, space, and labor is limiting as the number and scale of experiments increases. This loss in efficiency creates a logistical barrier to large-scale experiments in terms of the number of embryos that can be collected at given time points, even though a population of fish may actually be capable of producing enough embryos to support a given study. Further difficulties arise when experiments necessitate that embryos be at the same developmental stage for the purposes of treatment, manipulation, or analysis. To overcome these obstacles, we have developed a new method for the spawning and embryo collection of zebrafish that centers around the employment of an innovative, specialized breeding vessel. This technology capitalizes on the natural tendency of the fish to spawn in shallow water, a behavior that has been observed in nature [4], [5] and subsequently documented in domesticated fish in our laboratory [6]. The use of this apparatus effectively enables us to 1) collect very large numbers of embryos and 2) define precisely when those embryos will be fertilized.

## Materials and Methods

### Ethics Statement

The Institutional Animal Care and Use Committee at Children's Hospital Boston approved all experiments in which animals were used. (IACUC protocol # 08-11-1254R).

### Breeding Vessel Design and Architecture

**Figure 1:**
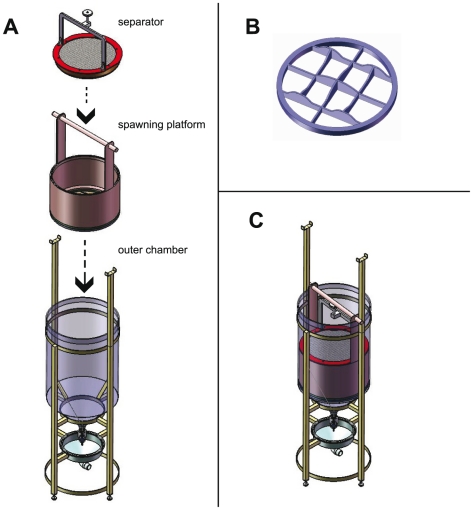
Architecture of the zebrafish breeding vessel. (A) The three primary components of the breeding vessel. (B) Framework of the bottom or “floor” of the spawning platform, showing variation in topography. (C) The breeding vessel, with all three primary components engaged and ready for operation.

The breeding vessel is comprised of three primary components: an outer chamber, a spawning platform, and a separator (Fig.1a). The outer chamber of the vessel is a 100 L cylindrical acrylic tank measuring 45.7 cm id ×81.3 cm tall supported by a stainless steel frame. The cylindrical bottom is fused to a funnel extending 20.3 cm creating a 41.6° angle with a 2.54 cm wide ball valve attached to the apex. The top of the four-legged frame contains two arms extending 56.5 cm above the chamber. The bottom portion of the frame consists of three 10.5 cm ×2.5 cm ×2.5 cm horizontal pegs that extend towards the middle of the frame and support the drainage funnel. The spawning platform is composed of opaque white polypropylene, and is made up of three separate pieces. The first is a hollow cylinder measuring 28.1 cm tall ×44.5 cm O.D. (outer diameter) ×0.635 cm thick. Two bands extend 33.5 cm high from the top end of the cylinder to support a horizontal dowel handle 2.54 cm diameter ×57.5 cm long that allow the platform to be lowered or raised within the outer chamber. Twelve 6.35 mm screws connect the bottom of the hollow cylinder to a mesh-forming ring. The mesh-forming ring, which is same O.D. as the hollow cylinder, is composed of six 0.635 cm bands of polypropylene, which are equally spaced and arranged in a crosswise pattern forming 90°angles. The bands at the bottom are equally flush while the tops are unevenly cut, creating mounds of higher elevation. A black polyethylene 3.18 mm mesh is lodged between the bottom of the hollow cylinder and the top of the mesh-forming ring. When fastened tightly and secured in place by small cable ties the mesh takes the shape of the forming ring, creating variable topography with a 12.7 mm difference in elevation from the lowest to the highest point of the mesh. This creates a bottom or “floor” of the platform that has undulating topography, with alternating high and low areas (Fig. 1b). The third major component of the breeding vessel is the separator, which is made up of two pieces of black polyethylene 3.18 mm mesh screwed to the top and bottom of an opaque white polypropylene spacer ring measuring 3.96 cm tall ×0.318 cm thick ×43.2 cm O.D. The top of the separator is characterized by having a flat ring 0.318 mm tall ×4 cm wide that contains a thumbscrew supported by two vertical arms, which rests centered at 21.6 cm above the top end of the separator.

### Breeding Vessel Operation

**Figure 2:**
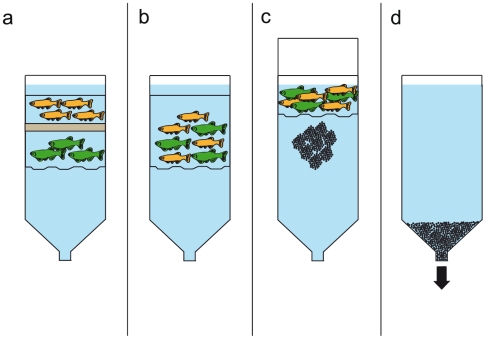
Schema depicting the operation of the breeding vessel, cross-sectional view. (A) The breeding vessel is filled with conditioned water, and fish are added to it so that female fish and male fish are contained within the spawning platform, below and above the separator, respectively. (B) The separator is removed and the male and female fish swim together in deep water. (C) The platform is raised within the outer chamber so that the male and female fish swim together and spawn in shallow water. The fertilized embryos fall through the floor of the spawning platform. (D) After the fish are removed from the breeding vessel, the fertilized embryos that have settled at the bottom of the outer chamber are collected.

During operation, the outer chamber is filled with pre-mixed, conditioned water from an off-system reserve tank (see description, below). The spawning platform is inserted into the chamber and pushed down so that its bottom is flush with where the cone portion of the chamber extends from the base of the cylinder. Pre-sorted, adult female zebrafish (colored green in Fig. 2a) are then transferred into the vessel, so that they are swimming within the spawning platform cylinder. The separator is then inserted into the apparatus and pushed down so that it is seated on the top lip of the platform, halfway down inside the chamber. The females are then all contained within the cylinder, underneath the bottom of the separator (Fig. 2a). Pre-sorted males (colored orange in Fig. 2a) are then added to the vessel, so that they are swimming inside the chamber, above the top of the separator. The total number of animals that may be added to the vessel should not exceed 250, as we found that holding fish (of the size used in these trials) at densities greater than 2.5 fish/L results in reduced performance (data not shown). Sex ratios were biased towards males in this set of trials to increase the rate of embryo production (having an excess of males ensures that all primed females in a given group will spawn as soon as the sexes are allowed to mingle). When embryos are desired, the separator is removed so that the males and females swim together in deep water (Fig. 2b). The platform is then immediately raised within the chamber to a level where the water depth inside the vessel is dramatically reduced (Fig. 2c). In this setting, the elevated areas of the undulated spawning platform floor are at or slightly above the water surface and the depressed areas are only 12.7 mm deep. Placing the spawning platform in this “shallow” physical arrangement immediately triggers spawning behavior in the fish [1]. Newly fertilized embryos fall through the openings of the mesh floor of the platform and rest at the bottom of the chamber. Spawning may be stopped at any time by removing the platform and the fish from the vessel. Embryos are collected by opening the ball valve at the bottom of the chamber and draining the water into a sieve (Fig. 2d).

### Animals

Two different populations of wild-type strain zebrafish (AB_1_ and AB_2_), and one population of a transgenic rps29 ribosomal mutant zebrafish (*rps29^hi2903Tg/+^*) were used in the breeding vessel validation and conventional cross comparison trials. A separate population of AB animals (AB_3_) was used in the shallow-deep sequence trials. AB fish were chosen for the trials because they are among the most common and important wild-type strains utilized in zebrafish research. The *rps29^hi2903Tg/+^* fish were selected to test the efficacy of the system on a mutant background with reduced genetic diversity. The fish from the AB_1_, AB_2_, AB_3,_ and *rps29^hi2903Tg/+^* populations were 24, 18, 5, and 10 months old at the time of the trials, respectively. The population size of each group was approximately 300 animals. The mean weight of individual fish in each population, for males and females (n = 60 for each sex, in each population), was 0.47±0.06 g and 0.62±0.07 g in the AB_1_ fish, 0.42±0.02 g and 0.60±0.03 g in the AB_2_ fish, 0.52±0.03 g and 0.66±0.01 g in the AB_3_ fish, and 0.36±0.02 g and 0.54±0.05 g in the *rps29^hi2903Tg/+^* fish.

### Animal Management and Conditioning

The fish were maintained in a 4500 L recirculating aquaculture system (Aqua Schwarz GmbH, Gottingen, Germany). The animals from each population used in the trials were housed in mixed sex groups on the system in multiple 9 L holding tanks at an approximate density of 6–7 fish/L. Photoperiod was 15 L:9D (light:dark), and the mean ranges for conductivity, pH, and temperature in the system were 1100–1300 µS, 7.5–8.0, and 26–29°C, respectively. Fish were fed to satiation 4X daily, 3X with *Artemia franciscana* nauplii (Artemia International LLC, Fairview, TX, USA), and 1X with NRD 400–600 Pellet (INVE Aquaculture Inc., Salt Lake City, UT, USA). Once a week, all fish from each population were removed from their tanks, pooled together and randomly redistributed back into tanks at the same densities to prevent dominance hierarchies potentially counterproductive to breeding success from being established [7].

### Water Production

The water used in the breeding trials and conventional cross comparisons was prepared by purifying municipal tap water by reverse osmosis and deionization. The resultant product was then reconstituted with Instant Ocean salt (Aquatic Ecosystems, Apopka, FL, USA) and sodium bicarbonate (Fisher Scientific, Waltham, MA, USA) to make “conditioned” water with a final conductivity of 1100–1300 µS and a pH 7.5–8.0. Water prepared in this fashion was stored in a 500-gallon reserve tank that is configured to independently supply both the recirculating aquaculture systems and specialized faucets at various sinks within the fish facility. Conditioned water was taken from faucets (and thus directly from the reserve tank) to fill the breeding vessel and conventional crossing cages.

### Breeding Vessel Validation Trials

Fish from the AB_1_, AB_2_, and *rps29^hi2903Tg/+^* populations were used in breeding vessel validation trials. Approximately 24 hours prior to each spawning event, 180 fish (100 males, 80 females) from a given population were sex segregated in the morning and returned back to the recirculating system (100 males in one 9 L tank, 80 females in two 9 L tanks) where they remained until set-up in the breeding vessel later in the afternoon. Eighteen hours prior to spawning, the outer chamber of the breeding vessel was filled with conditioned water (1100–1300 µS/ pH 7.5–8.0/ 26–29°C) from an off-system reserve tank and the fish were sequentially added to the chamber as previously described. In the morning on the following day, approximately 2 hours after the lights in the holding room came on, the breeding vessel was flushed with new, conditioned water from the off-system reserve tank to yield a 30% water change. We have found that changing a percentage of water in static breeding tanks (of any size or type) in the morning prior to releasing the fish improves spawning success, probably because it serves to reduce wastes built up in the water overnight as a result of the normal metabolism of the fish (Lawrence, unpublished data). The separator was removed immediately afterwards, allowing the males and females to swim together in deep water. The platform was then raised to the shallow water position and the fish were allowed to spawn for a 10-minute interval. The fish were then removed from the breeding vessel and the embryos were collected by opening the ball valve and draining the water in the vessel through a 200-micron mesh sieve. The collected embryos were measured volumetrically (1 mL = 600 embryos). After volumetric measurement, 100 embryos were randomly selected and reserved for 24 hours in a 50 mm petri dish to assess viability and developmental staging. The embryos that had developed normally up until that point were considered to be viable; those that had arrested or had undergone abnormal development were counted as non-viable. This procedure, which required one person to complete, was repeated three times, once per week, for each population. During the trials with the fish from the AB_2_ population, the procedure was timed, from start (sex segregation of test fish) to finish (collection of embryos).

### Conventional Cross Comparisons

Comparative spawning trials with the zebrafish from the AB_2_ population used in the breeding vessel trials were conducted in conventional 2.5 L static water spawning cages (Aqua Schwarz GmbH, Gottingen, Germany). 180 fish (100 males, 80 females) were sex-segregated as described above, in the morning, 24 hours prior to the trial. Approximately 18 hours prior to the trial, 40 cages were set up and filled with conditioned water from the off-system reserve tank and pre-sorted fish were added to them. Fish were added to spawning cages so that each contained either 2 males and 2 females or 3 males and 2 females. A divider was used to keep fish segregated in the cages overnight. The following morning, 18 hours after setup, (approximately 2 hours after the lights in the holding room came on) the tanks were arrayed onto the floor, and flushed with water from the off-system reserve tank, so that a 30% water change was achieved. Immediately afterwards, excess water was removed from the tanks to create a shallow water profile of approximately 12.7 mm deep. The dividers were then removed and the fish were allowed to spawn for one 10-minute interval. The fish were then removed from each spawning cage and all embryos were collected and measured volumetrically in the same manner described above. The embryos were assessed for viability and developmental staging in the same manner as described above. This procedure, which required two people to complete, was repeated three times, once per week, for this population. During each trial, the procedure was timed, from start (sex segregation of test fish) to finish (collection of embryos).

### Shallow-Deep Sequence Trials

In order to determine whether or not we could use the breeding vessel to generate multiple clutches of time-staged embryos with the same fish in one event, we conducted a set of trials where we repeatedly switched the position of the platform in the vessel from shallow to deep over a period of 150 minutes. Using the same setup methods described above, 90 fish (30 males, 60 females) from the AB_3_ population were added to the breeding vessel 18 hours prior to each trial. The sex ratio was biased towards females to help increase the duration of embryo production (an excess of females prolongs production because it generally takes longer for males to pair with all of the primed females in a given group). On the morning of the trial, after the chamber was given a 30% water change, the separator was removed, allowing the males and females to swim together in deep water. The platform was then raised to the shallow water position and the fish were allowed to spawn for a 10-minute interval. The platform was then immediately lowered the platform so that the fish were together in deep water. After 60 minutes in the deep water position, the platform was raised again to the shallow water position for another 10-minute interval. This sequence was repeated twice, so that in total, the fish were allowed to spawn in a sequence of five intervals: s1 (shallow from 0–10 minutes), d1 (deep from 10–70 minutes) s2 (shallow from 70–80 minutes), d2 (deep from 80–140 minutes), and s3 (shallow from 140–150 minutes). We collected the embryos spawned during each one of the five intervals by opening the ball valve at the end of the given interval and draining a few liters of water (containing the embryos spawned during the interval) from the vessel. Each time, the water lost during collection was immediately replaced with new conditioned water. All collected embryos from each interval were measured volumetrically in the same manner described above, except for intervals that produced less than 1 mL of embryos. In those instances, the embryos were counted by hand. 100 embryos from each interval were reserved and assessed for viability and developmental staging in the same manner as described above. In instances were an interval did not produce at least 100 embryos, the total number of embryos collected were reserved and assessed in this manner. This procedure, which required one person to complete, was repeated three times, once per week.

## Results

### Breeding Vessel Trials

**Figure 3:**
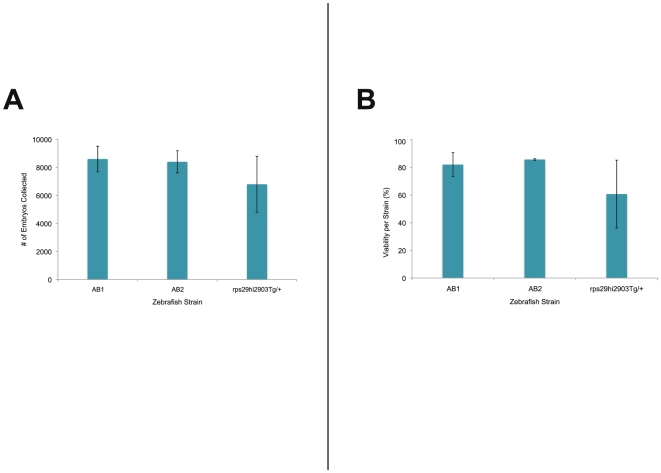
Quantitative assessment of embryo production and viability. (A) The average number of embryos produced in the breeding vessel during a 10 minute interval in separate populations of three zebrafish strains; (error bars, s.d.; n = 3). **(**B**)** The average viability of embryos produced in the breeding vessel during a 10 minute interval in separate populations of three zebrafish strains: (100 embryos sampled for each event; error bars, s.d.; n = 3).

The AB_1_, AB_2_, and *rps29^hi2903Tg/+^* fish produced mean per-interval clutch sizes of 8600±917, 8400±794, and 6800±1997 embryos, respectively (±.s.d., n = 3; Fig. 3a). The mean viability of the collected embryos was 0.82±0.09, 0.86±0.006 and 0.61±0.25 for the AB_1_, AB_2_, and *rps29^hi2903Tg/+^* fish, respectively (±.s.d., n = 3; Fig. 3b). Because the fish in each trial were only allowed to spawn within a 10-minute interval, 100% of the viable embryos collected from these events developed synchronously and were at the same developmental stage when we assessed them for viability 24 hours after collection.

### Conventional Cross Comparisons

AB_2_ fish set up in conventional crossing cages produced significantly fewer embryos than they did when they were set up in the breeding vessel (p<0.05; Table 1). Conventional crossing measures were also considerably less efficient in terms of the amount of time, labor, and space required to complete them. It took twice the number of staff performing this procedure to produce even those reduced quantities of embryos, and set up and break down times were also significantly higher (Table 1). Conventional crosses also required more than 5X the amount of space used the breeding vessel (Table 1).

**Table 1:** Comparison between conventional crosses and breeding vessel.

	Conventional Crosses (40)	Breeding Vessel (1)
Step	Average Time (minutes)	
Setup (day before)	77±6	22±2
Setup (morning of)	13±3	2±1
Breakdown	5±1	2±1
Embryo Collection	27±6	2±0.6
Total time	**122±7.6**	**29±2.6**
Space required (ft^2^)	16.7	2.92
Total embryos produced	4234±212^a^	8400±794^b^
Embryo viability (proportion)	0.87±0.02^a^	0.86±0.006^a^

Data for time, total embryos produced, and embryo viability are mean ± standard deviation. For embryo production and viability values, means with different superscript letters within each row are significantly different (Student's t-test, p<0.05).

### Shallow-Deep Sequence Trials

**Figure 4:**
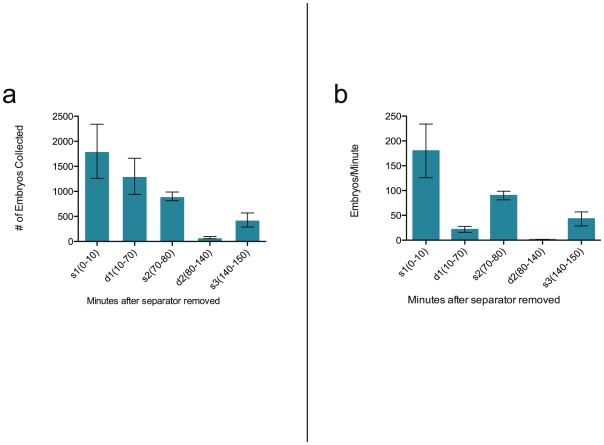
Embryo production over sequential shallow and deep water intervals. (A) The average number of embryos produced in the breeding vessel during five sequential intervals in one population of zebrafish; (error bars, s.d.: n = 3). s1 = 0–10 minutes post-release, shallow water, d1 = 10–70 minutes post-release, deep water, s2 = 70–80 minutes post-release, shallow water, d2 = 80–140 minutes post-release, deep water, s3 = 140–150 minutes post-release, shallow water. (B) The average rate of embryos produced per minute during five sequential intervals in one population of zebrafish; (error bars, s.d.: n = 3).

The AB_3_ fish spawned the greatest number of embryos during the first 10 minute, shallow water interval (s1), producing a mean clutch size of 1800±937 (±.s.d., n = 3; Fig. 4a). From that point on, the number of embryos produced per interval steadily decreased over the next three intervals. The fish produced mean clutch sizes of 1300±624, 900±150, and 76±36 during intervals d1, s2, and d2, respectively (±.s.d., n = 3; Fig. 4a). The number of embryos spawned during the last 10 minute shallow water interval, s3, which took place 140 minutes after the sexes were allowed to mingle, increased to a mean clutch size of 428±243 embryos (±.s.d., n = 3; Fig. 4a). The rate of embryo production was highest when the fish were held in the shallow water position within the breeding vessel, as the fish produced on average 180±94, 90±15, and 43±24 embryos/minute during s1, s2, and s3, respectively vs. 22±10, 1±1 during d1 and d2, respectively (±.s.d., n = 3; Fig. 4b). The mean viability of eggs collected during different intervals was 0.77±0.07, 0.78±0.08, 0.69±0.08, 0.63±0.19, 0.64±0.20, for s1, d1, s2, d2, and s3, respectively. The differences between these values were not statistically significant (p = 0.21, One-way ANOVA).

## Discussion

Our new system for spawning zebrafish is a major improvement over current methods, which have remained largely unchanged for nearly three decades, on a number of different levels. Our breeding vessel not only enables us to collect unprecedented quantities of embryos in single events, it also affords us with a level of control over the process not possible when using traditional equipment. By simply varying the depth profile of the water in the vessel from deep to shallow, we are able to greatly intensify the natural breeding behaviors of the fish. This allows us to achieve extremely high levels of production within very short windows of time, at spawning rates of up to 860 embryos/minute. It also enables us to collect multiple, timed clutches over a period of several hours, although spawning does not stop when male and female fish are together in deep water and the total number and rate of embryo production decreases steadily over time. Importantly, the rapidity of the process ensures that the embryos collected from such events are all developmentally synchronized and overcomes another limitation of traditional equipment. Finally, the new system makes great strides in efficiency. Indeed, our comparisons between the breeding vessel and conventional equipment show that while traditional crossing cages may also be used to generate similarly time-staged embryos, it is not possible to produce them in the same quantities that we are able to when using the breeding vessel without concomitantly and significantly increasing the number of fish, setups, space, and labor.

This is an important advance that has the potential to greatly accelerate the pace and scale of certain types of experiments conducted using the zebrafish model system. For example, the employment of our breeding vessel is now allowing us to make significant improvements in the throughput of the chemical genetic screening approaches that we employ in our laboratory [8]. Because we are now able to produce tens of thousands of age-matched embryos in single events with relative ease (especially when using multiple vessels), we have the ability to screen large chemical libraries in much shorter time frames. Our laboratory previously reported the results of a small molecule screen for suppressors of the *bmyb* mutant that took 4 months to complete when producing developmentally synchronized embryos at a rate of 5000 per week [9]. Our breeding vessel now makes it feasible that a screen of similar scale could be completed within a period of weeks as opposed to months.

The utility of this technological innovation will likely extend beyond simply making the process of screening small molecules in zebrafish more efficient. The approach should also serve to complement any existing [10], [11] and future efforts that capitalize on the amenability of the zebrafish to high throughput manipulation, analysis and automation.
